# Efficacy of Lysophosphatidylcholine as Direct Treatment in Combination with Colistin against *Acinetobacter baumannii* in Murine Severe Infections Models

**DOI:** 10.3390/antibiotics10020194

**Published:** 2021-02-17

**Authors:** Andrea Miró-Canturri, Rafael Ayerbe-Algaba, Manuel Enrique Jiménez-Mejías, Jerónimo Pachón, Younes Smani

**Affiliations:** 1 Clinical Unit of Infectious Diseases, Microbiology and Preventive Medicine, University Hospital Virgen del Rocío, 41013 Seville, Spain; amirocan93@gmail.com (A.M.-C.); ayerberafael@gmail.com (R.A.-A.); 2 Institute of Biomedicine of Seville (IBiS), University Hospital Virgen del Rocío, CSIC, University of Seville, 41013 Seville, Spain; pachon@us.es; 3 Department of Medicine, University of Seville, 41009 Seville, Spain

**Keywords:** lysophosphatidylcholine, colistin, direct treatment, *A. baumannii*

## Abstract

The stimulation of the immune response to prevent the progression of an infection may be an adjuvant to antimicrobial treatment. Here, we aimed to evaluate the efficacy of lysophosphatidylcholine (LPC) treatment in combination with colistin in murine experimental models of severe infections by *Acinetobacter baumannii*. We used the *A. baumannii* Ab9 strain, susceptible to colistin and most of the antibiotics used in clinical settings, and the *A. baumannii* Ab186 strain, susceptible to colistin but presenting a multidrug-resistant (MDR) pattern. The therapeutic efficacies of one and two LPC doses (25 mg/kg/d) and colistin (20 mg/kg/8 h), alone or in combination, were assessed against Ab9 and Ab186 in murine peritoneal sepsis and pneumonia models. One and two LPC doses combined with colistin and colistin monotherapy enhanced Ab9 and Ab186 clearance from spleen, lungs and blood and reduced mice mortality compared with those of the non-treated mice group in both experimental models. Moreover, one and two LPC doses reduced the bacterial concentration in tissues and blood in both models and increased mice survival in the peritoneal sepsis model for both strains compared with those of the colistin monotherapy group. LPC used as an adjuvant of colistin treatment may be helpful to reduce the severity and the resolution of the MDR *A. baumannii* infection.

## 1. Introduction

*Acinetobacter baumannii* is a Gram-negative bacterium with high clinical relevance owing to the increase in the number of nosocomial infections caused by this pathogen, as well as its ability to develop resistance to most antimicrobial agents used by physicians [1]. Treatment of *A. baumannii* infections, especially those caused by multidrug-resistant (MDR) strains, is a major concern. In many areas of the world that have a high prevalence of MDR *A. baumannii*, few options of treatment are present, and last resort treatments such as colistin are no longer effective in an increasing number of cases, leading to a 28-day mortality of 43% in hospitalized patients with bacteremia, ventilator-associated or hospital acquired pneumonia, or urosepsis [2]. The number of antibiotics approved by the Food and Drug Administration (FDA) cannot keep pace with the resistance mechanisms acquired by *A. baumannii*. Therefore, the development of new strategic antimicrobial therapeutic approaches, like the use of non-antibiotics in combination with one of the scarce but clinically relevant antibiotics, has become an urgent need.

A therapeutic alternative for the treatment of infections by MDR *A. baumannii* is immune system modulation to improve the infection clearance. We previously demonstrated the efficacy of lysophosphatidylcholine (LPC), a phospholipid involved in the recruitment and stimulation of immune cells [3,4,5,6], as a preemptive treatment in murine peritoneal sepsis and pneumonia experimental models by susceptible and MDR *A. baumannii* strains [7]. Of note, LPC preemptive treatment in combination with colistin, tigecycline or imipenem has improved the in vivo antibacterial activity of these antimicrobials in murine experimental peritoneal sepsis and pneumonia by drug-susceptible and MDR *A. baumannii* [8]. In the same line, preemptive LPC treatment in combination with ceftazidime has potentiated the in vivo antibacterial activity of ceftazidime in these severe infection models by MDR *Pseudomonas aeruginosa* [9]. Recently, Yadav et al. reported that LPC in vitro potentiated the effect of a nonbactericidal concentration of polymexin B against the growth of *Pseudomonas aeruginosa* and *Klebsiella pneumoniae* [10]. They found that a combination of LPC with polymexin B made pores in the bacterial membrane and caused the degradation of DnaK, the regulator of protein folding [10].

Currently, there are no data regarding the efficacy of the direct treatment with LPC in combination with colistin after infection by MDR *A. baumannii*, and whether this combined treatment can equalize or improve the preemptive LPC treatment in combination with colistin against MDR *A. baumannii*. Therefore, the aim of this study was to evaluate the efficacy of the direct treatment with LPC in combination with colistin in murine experimental models of peritoneal sepsis and pneumonia by drug-susceptible and MDR clinical isolates of *A. baumannii*.

## 2. Materials and Methods

### 2.1. Bacterial Strains

Drug-susceptible *A. baumannii* (Ab9) and MDR *A. baumannii* (Ab186) (resistant to imipenem, tigecycline, ciprofloxacin and ceftazidime) clinical strains were used in this study [8]. Both strains were susceptible to colistin with an MIC of 0.5 mg/L. The MIC of LPC against both strains was >8.000 mg/L [8]. Ab9 was recovered from surgical wound exudates, and Ab186 was recovered from blood cultures; the strains belong to ST297 and ST2 (international clone II), respectively [8,11].

### 2.2. Antimicrobial Agents and Reagents

A clinical formulation of colistin methanesulfonate (Promixin^®^, Bresso, Italia) was used. The anesthetic was 2:1 Ketamine hydrochloride^®^ (Pfizer, Madrid, Spain):Diazepam ^®^ (Roche, Madrid, Spain).

### 2.3. Animals

One hundred and seventy-five immunocompetent C57BL7/6 female mice weighing 18–20 g (Production and Experimentation Animal Center, University of Seville, Seville, Spain) were used. Animals were housed in regulation boxes and given free access to food and water. This study was carried out in strict accordance with the recommendations in the Guide for the Care and Use of Laboratory Animals [12]. The protocol was approved by the Committee on the Ethics of Animal Experiments of the University Hospital of Virgen del Rocío of Seville, Spain (approval 1556-N-16).

### 2.4. Experimental Murine Model of Peritoneal Sepsis

A previously characterized murine model of peritoneal sepsis caused by *A. baumannii* was used [8]. Briefly, animals were inoculated intraperitoneally (i.p.) with 0.5 mL of the 100% minimal lethal dose (MLD100) of Ab9 (5.9 log_10_ CFU/mL) or Ab186 (5 log_10_ CFU/mL), mixed 1:1 with 10% porcine mucin (Sigma, Madrid, Spain). LPC (Sigma, Madrid, Spain) and colistin treatments were administered 4 h after bacterial inoculation. Groups of mice were randomly ascribed to the following groups: (i) control (without treatment), (ii) LPC administered once i.p. at 25 mg/kg 4 h [7] after bacterial inoculation, (iii) colistin administered i.p. at 20 mg/kg/8 h for 72 h [8], (iv) the combination of colistin at 20 mg/kg/8 h with one dose of LPC at 25 mg/kg/d and (v) the combination of colistin at 20 mg/kg/8 h with two doses of LPC at 25 mg/kg/d (first and second at 4 and 28 h, respectively, after bacterial infection).

Mortality was recorded over 72 h. After the death or the euthanization of the mice by sodium thiopental (Zambon S.p.A., Vicenza, Italy) at the end of the experimental period, aseptic thoracotomies were performed, and blood samples were obtained by cardiac puncture. Spleens and lungs were aseptically removed and homogenized (Stomacher 80^®^; Tekman Co., London, UK) in 2 mL of sterile 0.9% NaCl solution. Tenfold dilution of the homogenized spleen and lungs and blood obtained by cardiac puncture were plated onto sheep agar for the quantitative cultures (to determine the log_10_ CFU/g of spleen and lungs and log_10_ CFU/mL of blood).

### 2.5. Experimental Murine Model of Pneumonia

A previously described experimental murine pneumonia model was used to evaluate the efficacy of LPC as monotherapy and in combination with colistin against Ab9 and Ab186 strains [8]. Briefly, the mice were anesthetized by 2:1 Ketamine hydrochloride:Diazepam, suspended vertically, and the trachea of each was then cannulated with a blunt-tipped metal needle. The feel of the needle tip against the tracheal cartilage confirmed the intratracheal location. A microliter syringe (Hamilton Co., Reno, NV, USA) was used for the inoculation of 50 µL of bacterial suspension (10 and 9 log_10_ CFU/mL for Ab9 and Ab186 strains, respectively), which had been grown for 24 h in LB broth at 37 °C and mixed at a 1:1 ratio with 0.9% NaCl solution containing 10% (wt/vol) porcine mucin. The mice remained in a vertical position for 3 min and then in a 30° position until they awakened. Treatment groups were similar to those used for the experimental model of peritoneal sepsis. After death or sacrifice of the mice at the end of the experimental period, aseptic thoracotomies were performed, blood was obtained by cardiac puncture and lungs were aseptically removed and homogenized. Quantitative data were obtained as described above to determine the log_10_ CFU/g of lungs and log_10_ CFU/mL of blood, and mice mortality was recorded over 72 h.

### 2.6. Statistical Analysis

Group data are presented as means ± standard errors of the means (SEM). Differences in the bacterial spleen, lung and blood concentrations (mean ± SEM log CFU per gram of tissue or per mL of blood) were assessed by analysis of variance (ANOVA) and the post hoc Dunnett test. Differences in mortality (%) and blood sterility (%) between groups were compared by the χ2 test. *p* values of <0.05 were considered significant. The SPSS (version 23.0; SPSS Inc., Armonk, NY, USA) statistical package was used.

## 3. Results

### 3.1. Efficacy of LPC in Combination with Colistin in a Murine Experimental Model of Peritoneal Sepsis

The efficacies of colistin and LPC in monotherapies and in combination against Ab9 and Ab186, expressed as survival and bacterial concentrations in spleen, lungs and blood, are shown in Table 1 and Table 2.

(i) Survival. Table 1 and Table 2 show that colistin alone and in combination with one and two doses of LPC increased mice survival compared with that of the control group for Ab9 and Ab186 (*p* < 0.05). In contrast, LPC in monotherapy did not reduce mice mortality. 

(ii) Bacterial clearance from spleen, lungs and blood. Table 1 and Table 2 show that monotherapy with colistin cleared Ab9 and Ab186 from the spleen, lungs and blood by 5.07 and 5.68 log_10_ CFU/g and 5.33 log_10_ CFU/mL (*p* < 0.05; Ab9), respectively, and 6.93 and 6.73 CFU/g and 6.7 log_10_ CFU/mL (*p* < 0.05; Ab186), respectively, compared with the levels of the control group. One dose of LPC in combination with colistin decreased spleen, lung and blood concentrations of Ab9 and Ab186 by 5.57 and 6.02 log_10_ CFU/g and 5.67 log_10_ CFU/mL (*p* < 0.05; Ab9), respectively, and 8.21 and 8.2 log_10_ CFU/g and 8.67 log_10_ CFU/mL (*p* < 0.05; Ab186), respectively, compared with the levels for the control group. In addition, the increase of the dose of LPC slightly increased the bacterial clearance. Two doses of LPC in combination with colistin reduced the bacterial burden in the spleen, lungs and blood by 6.13 and 6.72 log_10_ CFU/g and 6.74 log_10_ CFU/mL (*p* < 0.05; Ab9), respectively, and 9.57 and 8.88 log_10_ CFU/g and 8.81 CFU/mL (*p* < 0.05; Ab186), respectively, compared with the levels for the control group. Of note, one dose of LPC in combination with colistin decreased spleen, lung and blood concentrations of Ab9 and Ab186 by 5.84 and 5.86 log_10_ CFU/g and 6.28 log_10_ CFU/mL, respectively (*p* < 0.05; Ab9), and 8.9 and 8.6 log_10_ CFU/g and 9.07 log_10_ CFU/mL (*p* < 0.05; Ab186), respectively, compared with the levels for the LPC monotherapy group. Finally, two doses of LPC in combination with colistin decreased spleen, lung and blood concentrations for Ab9 and Ab186 by 6.4 and 6.56 log_10_ CFU/g and 6.74 log_10_ CFU/mL (*p* < 0.05; Ab9), respectively, and 10.26 and 9.28 log_10_ CFU/mL and 9.21 log_10_ CFU/mL (*p* < 0.05; Ab186), respectively, when compared with the levels for the LPC monotherapy.

### 3.2. The Efficacy of LPC in Combination with Colistin in a Murine Experimental Model of Pneumonia

The efficacies of colistin and LPC in monotherapies and in combination against Ab9 and Ab186, expressed as survival and bacterial concentrations in spleen, lungs and blood, are shown in Table 3 and Table 4.

(i) Survival. Table 3 and Table 4 show that colistin alone and in combination with one and two doses of LPC increased mice survival compared with that of the control group for Ab9 and Ab186 (*p* < 0.05). In contrast, LPC in monotherapy did not reduce mice mortality.

(ii) Bacterial clearance of lungs and blood. Table 3 and Table 4 show that monotherapy with colistin cleared Ab9 and Ab186 from the lungs and blood by 6.53 and 5.81 log_10_ CFU/g and mL (*p* < 0.05; Ab9), respectively, and 7.75 and 6.79 log_10_ CFU/g and mL (*p* < 0.05; Ab186), respectively, compared with the levels of the control group. One dose of LPC in combination with colistin decreased lung and blood concentrations of Ab9 and Ab186 by 6.76 and 6.08 log_10_ CFU/g and mL (*p* < 0.05; Ab9) respectively, and 8.1 and 7.17 log_10_ CFU/g and mL (*p* < 0.05; Ab186), respectively, compared with the levels for the control group. In addition, the increase of the dose of LPC slightly increased the bacterial clearance. Two doses of LPC in combination with colistin reduced the bacterial burden in lungs and blood by 7.74 and 6.64 log_10_ CFU/g and mL (*p* < 0.05; Ab9), respectively, and 8.56 and 7.33 CFU/g and mL (*p* < 0.05; Ab186), respectively, compared with the levels for the control group.

Finally, one and two doses of LPC in combination with colistin decreased the lung concentrations of Ab9 by 6.25 and 7.25 log_10_ CFU/g (*p* < 0.05) and Ab186 by 7.9 and 8.36 log_10_ CFU/g (*p* < 0.05), compared with the levels for the LPC monotherapy. Similar results were observed in blood, with a reduction of 5.4 and 5.95 log_10_ CFU/mL (*p* < 0.05; Ab9) and 6.74 and 6.9 log_10_ CFU/mL (*p* < 0.05; Ab186) compared with the levels for the LPC monotherapy.

## 4. Discussion

Previous studies from our group demonstrated that preemptive LPC monotherapy and LPC in combination with antibiotics such as colistin reduced bacterial tissue loads and bacteremia and increased mice survival in murine experimental models of severe infections by *A. baumannii* [7,8]. Even though LPC as preemptive monotherapy and in combination with colistin presented remarkable results, we hypothesized that it may be given as direct treatment in combination with colistin. 

Currently, colistin is among the last treatments available worldwide, being a last resort against MDR *A. baumannii* strains. Nevertheless, its therapeutic efficacy using optimal doses is limited, being effective just in 60% of patients infected with an MDR strain susceptible to colistin [13,14]. For that reason, two different clinical isolates were chosen, one drug-susceptible and one MDR, both susceptible to colistin. In the present study, monotherapy with colistin against drug-susceptible and MDR *A. baumannnii* strains significantly reduced bacterial concentrations in the spleen, lungs and blood and increased mice survival compared with the control group. However, it is important to highlight that colistin monotherapy presented a mortality rate of 75% in the case of the MDR strain in the peritoneal sepsis model. This result revealed a failure in the treatment with colistin, and the mice survival values are similar to and even higher than the rates obtained in a clinical practice when dealing with a colistin-susceptible strain with a highly resistant pattern. In accordance with our hypothesis, treatment with one or two doses of LPC in combination with colistin in a peritoneal sepsis model increased (without statistical difference) mice survival and reduced bacterial loads in tissues and blood, compared with colistin monotherapy. No differences were found between a single dose and multiple doses of LPC. It is noteworthy to mention that higher efficacy of the combination of LPC plus colistin was observed against the MDR strain Ab186, where survival rates were markedly increased. In the case of the pneumonia model, no differences were found in survival rates compared with colistin monotherapy, but decreases in lung and blood bacterial concentrations were observed.

Differences in bacterial concentrations were not due to different pharmacokinetic parameters between strains, since the MIC value of colistin for both strains is 0.5 mg/L. Different responses to the colistin treatment may be explained by immune responses caused by both strains. Indeed, Ab9 induced more TNF-alpha release than that of Ab186 [8]. Other studies reported by our group showed that a drug-susceptible *A. baumannii* strain induced more TNF-α and interleukin 6 releases than MDR and pan-drug-resistant *A. baumannii* clinical isolates [15,16]. In line with this hypothesis, increased lethality and severity of the infection by *A. baumannii* was observed when neutrophils were depleted, together with a delayed production of cytokines involved in neutrophil function, such as TNF-α, interleukin 1, keratinocyte chemoattractant protein (KC/CXCL1) and macrophage inflammatory protein (MIP-1) [17]. Neutrophils are essential players during *A. baumannii* infection and present an important role against sepsis and pneumonia infection [18,19]. It was reported that LPC blocks neutrophil deactivation during a murine cecal ligation and puncture model and increased the bactericidal activity of these immune cells [20]. Thus, the additive action of LPC to the antibiotic treatment may be due to the enhanced activity of neutrophils.

It is also believed that other mechanisms of action of LPC, independent of immune system regulation, are present, which might facilitate the action of antimicrobial agents. It was reported that LPC made pores in the membrane of *P. aeruginosa* and *K. pneumoniae* and degrades DnaK, the regulator of protein folding, which might potentiate the nonbactericidal effect of polymexin B [10]. In the same line, LPC interacts with the cytoplasmic membrane, induces the membrane depolarization and permeability of methicillin-resistant *Staphylococcus aureus* and potentiates the activity of ceftazidime [21]. Further investigations are needed to decipher the mechanism of action of LPC in combination with colistin against *A. baumannii*.

Interestingly, direct treatment with LPC in combination with colistin presents similar efficacy to preemptive treatment with LPC in combination with colistin against the MDR Ab186 strain. A reduction of the bacterial burden in spleen and lungs of around 2 log_10_ CFU/g in a murine model of peritoneal sepsis and pneumonia models compared with LPC in combination with colistin preemptive treatment was observed [8]. This comparison increases the interest towards LPC as a future adjuvant therapy with colistin, which may reduce the phenomenon of resistance to antibiotics [22].

## 5. Conclusions

The present study suggests that treatment with LPC in combination with colistin after bacterial infection improves the in vivo antibacterial activity in murine experimental models of peritoneal sepsis and pneumonia by MDR *A. baumannii* by further reducing bacterial loads in tissues and blood and increasing mice survival.

## Figures and Tables

**Table 1:** The therapeutic effect of one or two doses of LPC in combination with colistin in a murine model of peritoneal sepsis with *A. baumannii* Ab9.

Treatment	*n*	Spleen (log_10_ CFU/g)	Lung (log_10_ CFU/g)	Blood (log_10_ CFU/mL)	Mortality (%)
CTL	10	9.55 ± 0.99	9.85 ± 0.72	8.59 ± 0.04	100
LPC	8	9.82 ± 0.08	9.69 ± 0.91	9.20 ± 0.04 ^a^	100
CST	8	4.48 ± 0.30 ^a,b^	4.17 ± 0.29 ^a^	3.26 ± 0.40 ^a,b^	25 ^a^
LPC1 + CST	8	3.98 ± 0.66 ^a,b^	3.83 ± 0.65 ^a^	2.92 ± 0.58 ^a,b^	0 ^a^
LPC2 + CST	8	3.42 ± 0.50 ^a,b^	3.13 ± 0.46 ^a^	1.85 ± 0.38 ^a,b^	0 ^a^

CTL, control (no treatment); LPC, lysophosphatidylcholine; CST, colistin; LPC1, one dose of lysophosphatidylcholine; LPC2 two doses of lysophosphatidylcholine; *n* = number of mice. ^a^ *p* < 0.05 compared to the controls. ^b^ *p* < 0.05 compared to the LPC group.

**Table 2:** The therapeutic effect of one or two doses of LPC in combination with colistin in a murine model of peritoneal sepsis with *A. baumannii* Ab186.

Treatment	*n*	Spleen (log_10_ CFU/g)	Lung (log_10_ CFU/g)	Blood (log_10_ CFU/mL)	Mortality (%)
CTL	13	9.79 ± 0.06	9.63 ± 0.13	8.89 ± 0.03	100
LPC	8	10.48 ± 0.03	10.03 ± 0.03	9.29 ± 0.03	100
CST	8	2.86 ± 1.54 ^a,b^	2.90 ± 1.57 ^a,b^	2.19 ± 1.63 ^a,b^	75
LPC1 + CST	12	1.58 ± 0.48 ^a,b^	1.43 ± 0.54 ^a^	0.22 ± 0.21 ^a,b^	0 ^a,b^
LPC2 + CST	12	0.22 ± 0.29 ^a,b^	0.75 ± 0.32 ^a^	0.08 ± 0.12 ^a,b^	0 ^a,b^

CTL, control (no treatment); LPC, lysophosphatidylcholine; CST, colistin; LPC1, one dose of lysophosphatidylcholine; LPC2 two doses of lysophosphatidylcholine; *n* = number of mice. ^a^ *p* < 0.05 compared to the controls. ^b^ *p* < 0.05 compared to the LPC group.

**Table 3:** The therapeutic effect of one or two doses of LPC in combination with colistin in a murine model of pneumonia with *A. baumannii* Ab9.

Treatment	*n*	Lung (log_10_ CFU/g)	Blood (log_10_ CFU/mL)	Mortality (%)
CTL	8	9.64 ± 0.55	7.95 ± 0.83	87.5
LPC	8	9.13 ± 0.28	7.27 ± 0.04 ^a^	100
CST	8	3.11 ± 1.18 ^a,b^	2.14 ± 0.57 ^a,b^	12.5 ^a,b^
LPC1 + CST	8	2.88 ± 1.12 ^a,b^	1.87 ± 0.6 ^a,b^	12.5 ^a,b^
LPC2 + CST	8	1.90 ± 1.13 ^a,b^	1.31 ± 0.80 ^a,b^	12.5 ^a,b^

CTL, control (no treatment); LPC, lysophosphatidylcholine; CST, colistin; LPC1, one dose of lysophosphatidylcholine; LPC2 two doses of lysophosphatidylcholine; *n* = number of mice. ^a^ *p* < 0.05 compared to the controls. ^b^ *p* < 0.05 compared to the LPC group.

**Table 4:** The therapeutic effect of one or two doses of LPC in combination with colistin in a murine model of pneumonia with *A. baumannii* Ab186.

Treatment	*n*	Lung (log_10_ CFU/g)	Blood (log_10_ CFU/mL)	Mortality (%)
CTL	8	9.21 ± 0.45	7.76 ± 0.39	100
LPC	8	9.01 ± 0.07	7.33 ± 0.03	100
CST	8	1.66 ± 0.49 ^a,b^	0.97 ± 0.30 ^a,b^	0 ^a,b^
LPC1 + CST	8	1.11 ± 0.54 ^a,b^	0.59 ± 0.29 ^a,b^	0 ^a,b^
LPC2 + CST	8	0.65 ± 0.43 ^a,b^	0.43 ± 0.28 ^a,b^	0 ^a,b^

CTL, control (no treatment); LPC, lysophosphatidylcholine; CST, colistin; LPC1, one dose of lysophosphatidylcholine; LPC2 two doses of lysophosphatidylcholine; *n* = number of mice. ^a^ *p* < 0.05 compared to the controls. ^b^ *p* < 0.05 compared to the LPC group.

## Data Availability

Not applicable.
